# MCUR1 facilitates epithelial-mesenchymal transition and metastasis via the mitochondrial calcium dependent ROS/Nrf2/Notch pathway in hepatocellular carcinoma

**DOI:** 10.1186/s13046-019-1135-x

**Published:** 2019-03-25

**Authors:** Mingpeng Jin, Jiaojiao Wang, Xiaoying Ji, Haiyan Cao, Jianjun Zhu, Yibing Chen, Jin Yang, Zheng Zhao, Tingting Ren, Jinliang Xing

**Affiliations:** 1 State Key Laboratory of Cancer Biology and Experimental Teaching Center of Basic Medicine, Fourth Military Medical University, 169 Changle West Road, Xi’an, 710032 China; 2 Center of Genetic & Prenatal Diagnosis, First Affiliated Hospital, Zhengzhou University, Zhengzhou, 450052 China; 3 Institute of Preventive Genomic Medicine, School of Life Sciences, Northwest University, Xi’an, 710069 China; 4 Third Department of Medical Oncology, Shaanxi Provincial Cancer Hospital, Xi’an, 710061 China

**Keywords:** Mitochondrial calcium uniporter regulator 1, Hepatocellular carcinoma, EMT, Metastasis, Notch 1

## Abstract

**Background:**

Mitochondrial Ca^2+^ plays a critical role in tumorigenesis, including cell proliferation and metastasis. Mitochondrial calcium uniporter regulator 1 (MCUR1) has been shown to be frequently upregulated in HCC and promote cancer cell survival. However, whether MCUR1 is involved in the metastasis of HCC and its underlying mechanisms remain unknown.

**Methods:**

The effect of MCUR1 expression on epithelial-mesenchymal transition (EMT) in HCC cells was first evaluated by immunofluorescent staining and Western blot. Then, in vitro invasion and in vivo metastasis assays were used to evaluate the function of MCUR1 in HCC metastasis. The underlying mechanism has also been explored by investigating the effect of MCUR1 on ROS/Nrf2/Notch1 pathway.

**Results:**

MCUR1 expression was significantly higher in HCC with metastasis and associated with tumor progression. MCUR1 promoted in vitro invasion and in vivo metastasis of HCC cells by promoting EMT via Snail. Mechanistically, MCUR1-mediated mitochondrial Ca^2+^ signaling promoted the EMT of HCC cells by activating ROS/Nrf2/Notch1 pathway. Inhibition of ROS production, mitochondrial Ca^2+^ uptake, Nrf2 expression or Notch1 activity significantly suppressed MCUR1-induced EMT of HCC cells. In addition, treatment with the mitochondrial Ca^2+^-buffering protein parvalbumin significantly inhibited ROS/Nrf2/Notch pathway and MCUR1-induced EMT and HCC metastasis.

**Conclusions:**

Our study provides evidence supporting a metastasis-promoting role for MCUR1-dependent mitochondrial Ca^2+^ uptake in HCC. Our findings suggest that MCUR1 may be a potential therapeutic target for HCC treatment.

**Electronic supplementary material:**

The online version of this article (10.1186/s13046-019-1135-x) contains supplementary material, which is available to authorized users.

## Background

Hepatocellular carcinoma (HCC) is one of the most common malignant tumors worldwide. The HCC population in Asia and Africa accounts for the majority of all cases [1]. HCC has the characteristics of slow onset, high aggressiveness and rapid growth [2]. Despite improvements in comprehensive treatment regimen and considerable progress made in understanding its epidemiology, etiology, biology, diagnosis and treatment, the long-term prognosis of patients with HCC remains poor. Approximately 90% of cancer death are caused by metastasis, a complicated process involving tumor cell motility, intravasation, circulation, extravasation and growth in new tissues and organs [3]. The increased motility and invasive properties of metastatic tumor cells are reminiscent of events that occur during epithelial-mesenchymal transition (EMT). EMT is a process in which epithelial cells lose their cell polarity and cell-cell adhesion, and then gain a mesenchymal phenotype with migratory and invasive properties. EMT has been proposed as a vital mechanism for epithelial cancer cells to acquire a malignant phenotype, especially invasion and metastasis [4]. EMT activation is usually initiated and controlled by cellular signals that respond to extracellular cues, such as TGF-β, Wnt, Notch, and Hedgehog signaling pathways. However, the mechanism by which tumors induce EMT to facilitate invasion and metastasis remains largely unknown.

The calcium signal is a key mechanism well suited to the rapid translation of signals from the tumor microenvironment into cellular responses. In addition to its important roles in the growth of the primary tumor, calcium signaling is also important in the context of cancer cell migration and invasion [5]. Calcium-dependent migration and invasion has been observed in prostate and breast cancer cells [6, 7]. A number of well-known molecular players in cellular Ca^2+^ homeostasis, such as the constituents of store-operated Ca^2+^ entry and calcium release-activated calcium channel proteins, have been implicated in the process of cancer cell metastasis and migration.

As the “power factory” of the cell, mitochondria have also been generally considered to regulate the intracellular calcium homeostasis. Mitochondrial calcium uptake is believed to be essential in regulating cellular signaling events, energy status, reactive oxygen species (ROS) production and survival [8]. The uptake of Ca^2+^ by mitochondria depends on mitochondrial Ca^2+^ uptake channel (mitochondrial calcium uniporter, MCU) and its regulatory subunits, such as MICU1 (mitochondrial calcium uptake 1) and MCUR1 (mitochondrial calcium uniporter regulator 1) [9–11]. Previous studies have demonstrated that MCU complex and its regulatory proteins are frequently dysregulated in several types of cancer, including breast, prostate, ovarian and colorectal cancer [7, 12–14]. Moreover, the expression aberration of these proteins facilitates proliferation, migration, invasion and apoptotic resistance of cancer cells and is frequently associated with poor prognosis of cancer patients [15].

In our previous study, we have found that MCU expression is significantly elevated in HCC cells and MCU-dependent mitochondrial Ca^2+^ uptake promotes ROS production, cell metastasis and poor prognosis of HCC patients [15]. Moreover, we have also found that MCUR1-mediated mitochondrial calcium signaling promotes HCC cell survival via ROS signaling [16]. These data suggest that MCUR1 may be involved in the metastasis and invasion of HCC. However, this hypothesis remains to be tested and the underlying mechanisms need further investigation.

In the present study, we systematically investigated the effects of MCUR1 on HCC metastasis and the underlying mechanisms. We have demonstrated that MCUR1-mediated mitochondrial calcium signaling triggers ROS generation and subsequent Notch signaling pathway, which contributes to the EMT of HCC cells and poor prognosis of patients. Our data provide novel evidence supporting MCUR1 as a potential promising therapeutic target for the treatment of HCC patients.

## Methods

### Antibodies and reagents

Antibodies used in this study were listed in Additional file 1: Table S1. The mitoROS scavenger MitoTEMPO, Nrf2 activator OPZ, Notch1 inhibitor DAPT, H_2_O_2_, histamine were purchased from Sigma-Aldrich (St Louis, MO, USA).

### Cell culture and tissue

Human HCC cell lines BEL7402 and MHCC97L were from the Shanghai Cell Bank of the Chinese Academy of Sciences (Shanghai, China). HCC cell lines were authenticated using short tandem repeat DNA testing by the FMMU Center for DNA Typing in 2018. And all cell lines were routinely cultured. Human HCC tissue samples without metastasis (*n* = 74) or with metastasis (*n* = 63) from surgical HCC patients were obtained in Xijing Hospital affiliated with Fourth Military Medical University, Xi’an, China, with signed informed consents. All tissues were assessed by H&E staining to select suitable regions for further examination. The latest follow-up date was January 2016 and the median follow-up duration was 28.5 months (ranging from 2.4 to 85.6 months).

### Knockdown and forced expression for the target genes

A small hairpin RNA (shRNA) specifically targeting the human MCUR1 mRNA sequence (5′-AAGGACAUCGUCUACAAAGAU-3′) was cloned into the vector of pSilencer™ 3.1-H1 puro (Ambion). For overexpression of MCUR1, the cDNA of MCUR1 was cloned into the vector of pcDNA™3.1(+) (Invitrogen). All siRNAs were synthesized by GenePharma (Shanghai, China) and the sequences were provided in Additional file 1: Table S2. The cDNA of the Ca^2+^ binding protein parvalbumin (PV) was cloned into pAc1GFP1-Mito vector using primers listed in Additional file 1: Table S2 to establish PV-Mito-green fluorescent protein and PV-Mito construct. The pAd-Easy adenovirus system was used for generation of recombinant adenoviruses carrying mitochondria-targeted PV (Ad-PV-Mito).

### Immunofluorescence staining assay

Cells grown in 15-mm coverglass-bottom dish (NEST, Wuxi, China) were fixed with 4% paraformaldehyde in phosphate-buffered saline (PBS) with 0.2% Triton. Cells were then blocked for 1 h with 2% bovine serum albumin followed by incubation with primary antibody overnight at 4 °C and then with fluorophore-conjugated secondary antibody (Jackson Immunoresearch, West Grove, PA). Cells were examined with a confocal laser scanning microscope FV1000 (Olympus).

### Western blot analysis

Protein expression levels in HCC cells were examined by Western blot as our previous description [17] . The antibodies and their dilutions were listed in the Additional file 1: Table S1.

### Scratch wound healing assay

Cells were seeded in six-well plates and cultured to almost total confluence in 24 h, and then a wound was scratched in each well using a 10-μl pipette tip. Cells were washed with PBS for several times and then incubated in culturing medium without serum. Wound closure was monitored at 0 and 48 h on a Nikon Eclipse TS100 microscope. The distance of cell migration at 0 and 48 h after scratching was evaluated. Migration rate (%) was calculated as (Migrated distance at indicated time - initial distance) / (initial distance) × 100%.

### Transwell assays

Cell migration and invasion ability was assessed using transwell assay. For cell invasion assays, transwell chambers were coated with Matrigel. The upper chamber was seeded with 3 × 10^4^ cells suspended in 500 μl of serum-free medium, while the lower chamber was filled with 20% FBS medium. After 24 h incubation, the cells in the upper chamber were carefully removed with a cotton swab. The chamber was then immersed in 4% paraformaldehyde for 30 min and stained with 0.1% crystal violet for 5 min. The invasive cells attaching to the lower surface of the membrane were counted under microscopy. For cell migration assays, the procedure was similar to that for the invasion assays without Matrigel coating.

### Nude mice xenograft metastasis model

For in vivo metastasis assay, five-week-old and weighing 18-20 g each nude mice (BALB/c) were randomly divided into subgroups (five mice per group). Cells (2.0 × 10^6^ for each mouse) were resuspended in 50 μL PBS and orthotopically injected into the left hepatic lobe of nude mice (male). Eight weeks later, mice were sacrificed under anesthesia. The liver and lung were resected and fixed with 4% paraformaldehyde. Serial 5-μm sections were stained with H.E. for histopathological examination. Metastasis lesions from 10 random high-power fields were counted. Housing and all other procedures were performed according to protocols approved by the Animal Experimentation Ethics Committee of the Fourth Military Medical University. All animals received human care and study protocols were complied with the institution’s guidelines.

### Immunohistochemical (IHC) staining

Four-μm-thick tissue sections were cut from human HCC tissue blocks and xenograft tumor nodes. IHC was performed as previously described [17]. The intensity and extent of immunostaining were evaluated for all samples under double-blinded conditions as previously described [17].

### Measurement of mitochondrial Ca^2+^

For measurement of basal mitochondrial Ca^2+^, cells were transiently transfected with the plasmid carrying mitochondrial matrix-targeted inverse pericam, which was defined as mitopericam (kindly provided by Dr. Atsushi Miyawaki from RIKEN Brain Science Institute, Japan). Then cells were examined with a confocal laser scanning microscope FV1000 (Olympus, Tokyo, Japan). For measurement of dynamic mitochondrial Ca^2+^, histamine (10 μM) was added after 30 s of baseline recording, 380 and 490 nm excitation filters were used together with a 540 nm emission filter and images were recorded every 3 s.

### Detection of reactive oxygen species

Fluorescence probe DCFH-DA (Beyotime, Beijing, China) was used to detect cellular reactive oxygen species (ROS). The fluorescence intensity was assessed by flow cytometry with an excitation wavelength of 488 nm and an emission wavelength of 535 nm. The fluorescence probe mitoSOX (Invitrogen) was used to detect mitochondrial reactive oxygen species (mitoROS) under microscopy (FluoView 1000). Images were captured and analyzed by ImagePro image analysis software (Media Cybernetics, Silver Spring, MD, USA).

### Statistical analysis

Experiments were repeated three times, where appropriate. Data represent mean ± SD. SPSS 17.0 software (SPSS, Chicago, IL) was used for all statistical analyses and *P* < 0.05 was considered significant. Unpaired t tests were used for comparisons between 2 groups where appropriate. Correlations between measured variables were tested by Spearman’s rank correlation analyses.

## Results

### MCUR1 promoted EMT of HCC cells

**Fig. 1:**
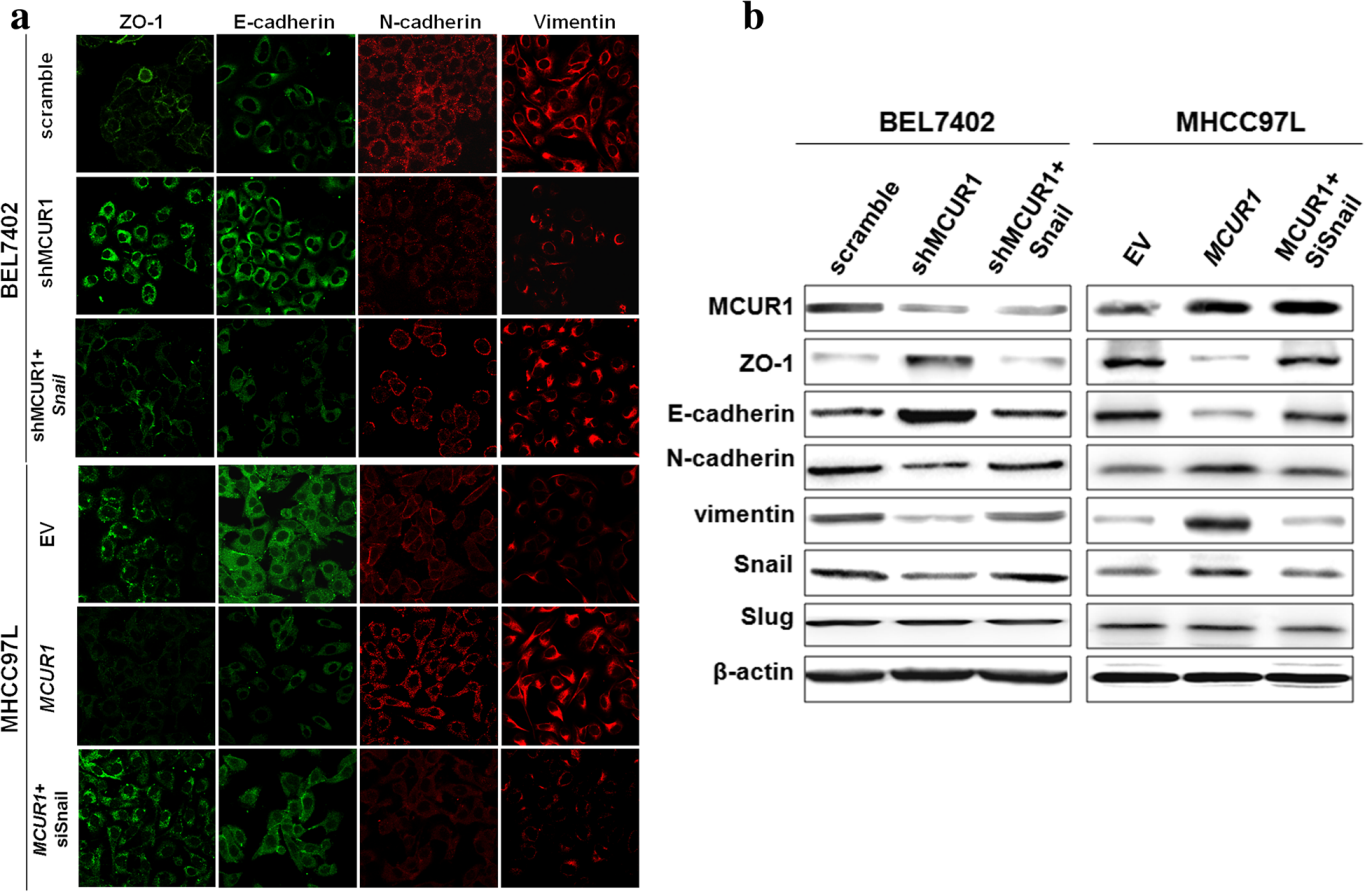
MCUR1 promoted EMT of HCC cells. **a** Immunofluorescent image of epithelial markers (ZO-1 and E-cadherin) and mesenchymal markers (N-cadherin and Vimentin) in HCC cells with different MCUR1 expression levels. Cells were transfected with siRNA or expression vector for 48 h. Scramble: vector encoding control shRNA; shMCUR1 vectors encoding short hairpin RNA (shRNA) against MCUR1. EV: Empty Vector; MCUR1: expression vectors encoding MCUR1; Snail, Snail expression vector; siSnail, siRNA against Snail. **b** Western blot analysis of ZO-1, E-cadherin, N-cadherin, Vimentin, Snail and Slug in HCC cells with different MCUR1 expression levels

As previously described [16], to more effectively investigate the phenotype changes, cells with the relatively high or low expression of MCUR1 was respectively selected to establish the cell models with knockdown or forced expression of MCUR1. Our results showed that MCUR1 knockdown increased the expression of epithelial markers (ZO-1 and E-cadherin) and decreased the expression of the mesenchymal markers (N-cadherin and Vimentin), whereas MCUR1 overexpression exhibited an opposite effect (Fig. 1a). Western blot analysis further validated the observations in IF assay (Fig. 1b). Moreover, the expression of EMT-related transcriptional factor Snail but not Slug was significantly affected by MCUR1 (Fig. 1b). Furthermore, Snail overexpression significantly compensated MCUR1 knockdown-induced repression of EMT, while Snail knockdown significantly inhibited the MCUR1-induced EMT of HCC cells (Fig. 1a, b and Additional file 2: Figure S1f).

As shown in Additional file 2: Figure S1a, our results showed that HCC cells with MCUR1 knockdown exhibited the morphological change from mesenchymal to epithelial and had a significantly decreased basal mitochondrial Ca^2+^ ([Ca^2+^]_m_) when compared with the control cells. In contrast, HCC cells with MCUR1 overexpression displayed opposite results (Additional file 2: Figure S1b and c). We further investigated the effect of MCUR1 on the capability of mitochondrial Ca^2+^ uptake. Histamine (InsP3-linked agonist) was used to rapidly elevate the intracellular Ca^2+^ ([Ca^2+^]_c_) and thus trigger the mitochondrial Ca^2+^ uptake. Our data indicated that the capability of mitochondrial Ca^2+^ uptake was significantly inhibited in HCC cells with MCUR1 knockdown, whereas it was clearly increased in those with MCUR1 overexpression.

Mitochondria serves as a major source of intracellular ROS (total ROS), which are commonly increased in cancer cells and drives a series of events such as the second messenger in tumor metastasis. Therefore, we determined whether the MCUR1 mediated mitochondrial Ca^2+^ uptake would affect ROS production. As shown in Additional file 2: Figure S1d and e. MCUR1 knockdown significantly decreased the level of total ROS, whereas overexpression of MCUR1 increased the total ROS level in HCC cells. When the level of mROS, which is the main contribution of total ROS, was measured by mitochondrial superoxide indicator MitoSOX Red, similar results were observed. These data indicate that the MCUR1 expression considerably affects the production of ROS in HCC cells.

### MCUR1 promoted in vitro invasion and migration of HCC cells by snail-mediated EMT

**Fig. 2:**
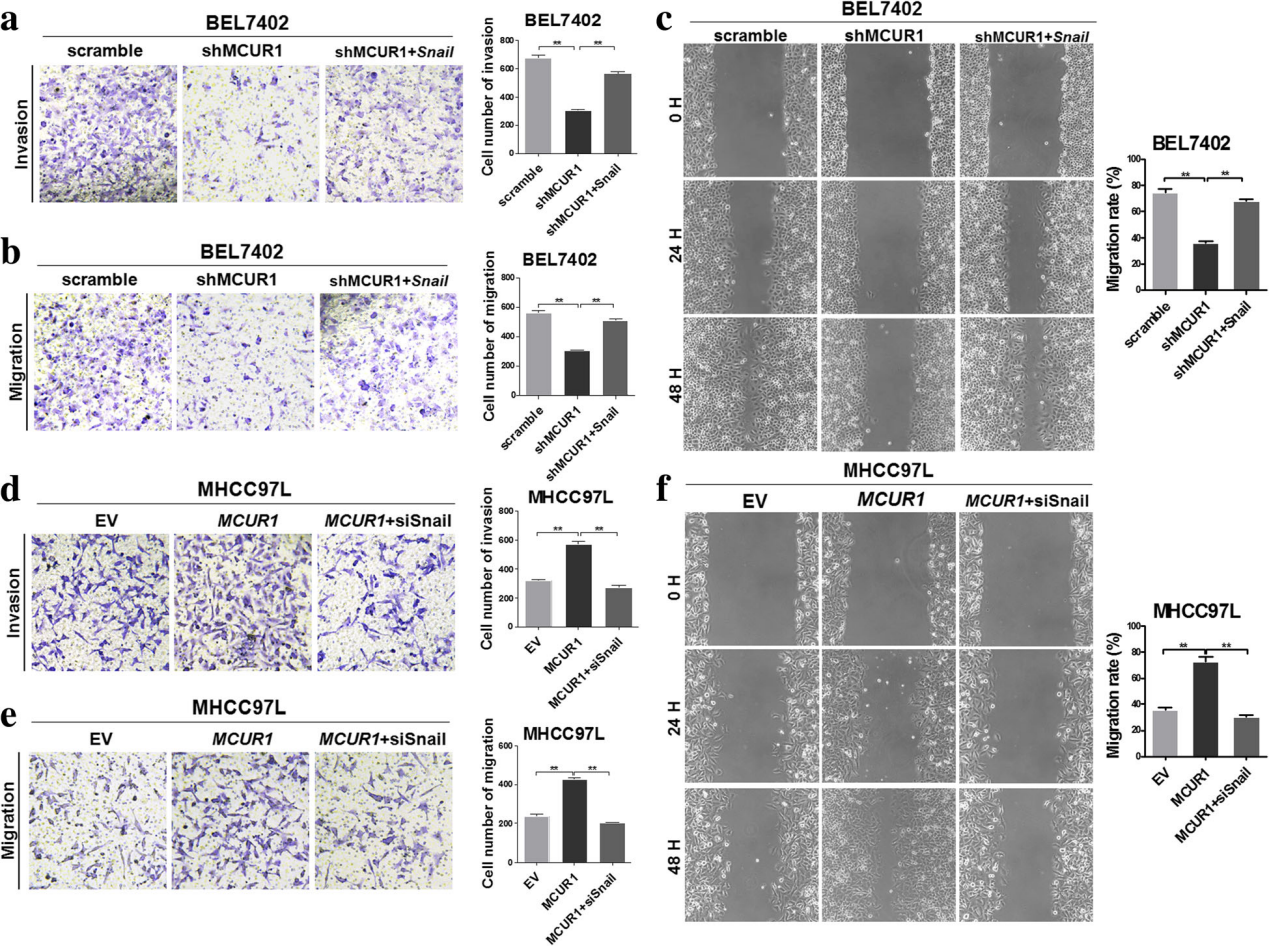
MCUR1 promoted in vitro invasion and migration of HCC cells by snail-mediated EMT. Transwell assay for ability of invasion (**a**, **d**) and migration (**b**, **e**) in stably transfected HCC cells with treatment as indicated. **c**, **f** Representative images of wound-healing assay and corresponding statistical analysis in stably transfected HCC cells with treatment as indicated. Data shown are the mean ± SD from three independent experiments. * *P* < 0.05; ** *P* < 0.01

Transwell and scratch assays demonstrated that MCUR1 knockdown significantly decreased the migration and invasion capabilities of HCC cells, which could be reversed by up-regulating the expression of Snail (Fig. 2a, b and c). In contrast, MCUR1 overexpression promoted the migration and invasion of HCC cells, which could be reversed by Snail knockdown (Fig. 2d, e, and f). Furthermore, we found the similar phenomenon that mice in MCUR1 knockdown group exhibited significant less intrahepatic metastasis and distal lung metastasis than control group. In contrast, MCUR1 overexpression exhibited a promoting effect on HCC intrahepatic metastasis and distal lung metastasis. And these effects were reversed by forced expression of Snail or interference of Snail expression (Additional file 3: Figure S2a, b and c).

### MCUR1 facilitated EMT and HCC metastasis in vivo

**Fig. 3:**
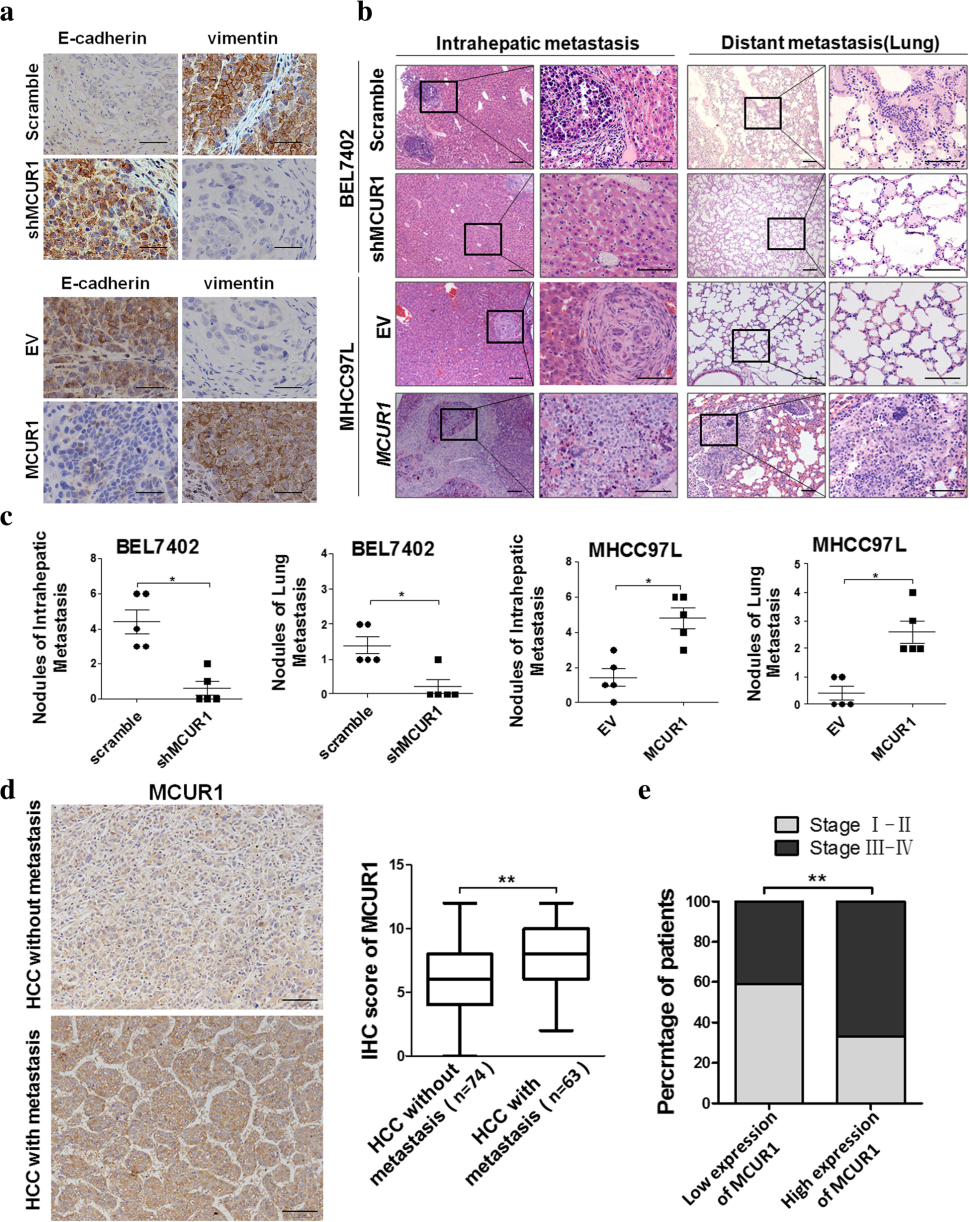
MCUR1 facilitates epithelial-mesenchymal transition and HCC metastasis. **a** Representative immunohistochemical staining images of E-cadherin and Vimentin in the orthotopic transplantation nude mice model of HCC metastasis. **b** Histological analyses of intrahepatic and lung metastatic nodules from HCC metastasis nude mice model by hematoxylin and eosin (H&E) staining. Images showing representative H&E staining of liver and lung tissue samples from the different experimental groups (*n* = 5 /group). **c** The number of intrahepatic and lung metastasis nodules was quantified in H.E. sections. **d** Representative immunohistochemical staining images and IHC score of MCUR1 in HCC without metastasis (*n* = 74) and HCC with metastasis (*n* = 63). **e** MCUR1 expression positively correlated with tumor progression of HCC patients. Data shown are the mean ± SD from three independent experiments. * *P* < 0.05; ** *P* < 0.01

To evaluate the role of MCUR1 in tumor EMT and metastasis in vivo, we established the orthotopic transplantation model of HCC metastasis in nude mice. The metastasis foci were clearly identified under microscope. However, we did not find the macroscopic metastases in liver and lung tissues possibly due to short time from cell transplantation to mice sacrifice. Immunohistochemical (IHC) assays of xenograft tumors demonstrated that MCUR1 knockdown induced higher expression of the epithelial marker E-cadherin and lower expression of the mesenchymal marker Vimentin, whereas MCUR1 overexpression had an opposite effect (Fig. 3a). Furthermore, mice in MCUR1 knockdown group exhibited significantly less intrahepatic metastasis and distal lung metastasis than control group. In contrast, MCUR1 overexpression exhibited a promoting effect on HCC intrahepatic metastasis and distal lung metastasis (Fig. 3b and c).

### MCUR1 expression was associated with HCC metastasis and clinical stage of patients with HCC

Next, we evaluated the expression of MCUR1 in HCC with (*n* = 64) or without (*n* = 74) intrahepatic metastasis using IHC staining. Our results showed that MCUR1 was more frequently upregulated in HCC tissues with metastasis than those without metastasis (Fig. 3d). Moreover, MCUR1 expression was positively correlated with TNM stage of HCC (Fig. 3e).

### MCUR1 facilitated ROS-induced Nrf2 nuclear translocation to activate the Notch pathway

**Fig. 4:**
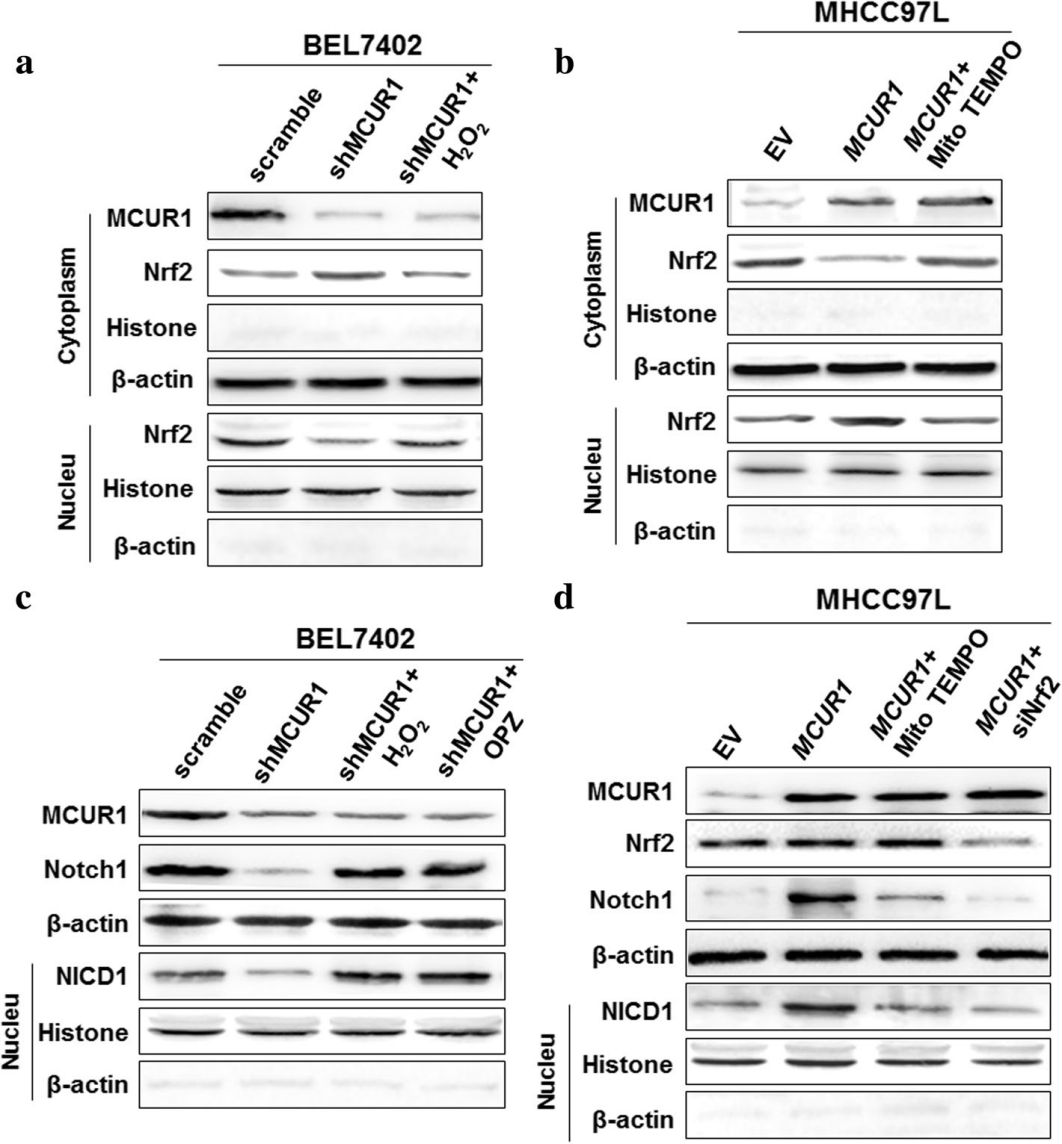
MCUR1 facilitates ROS-induced Nrf2 nuclear translocation to activate the Notch pathway. **a**, **b**, **c**, **d** Western blot analysis for expression levels of Nrf2, Notch1and NICD1 in stably transfected HCC cells with treatments as indicated

Our results had showed that MCUR1 considerably increased mitochondrial Ca^2+^ ([Ca^2+^]_m_) and ROS in HCC cells. And previous evidence has shown that Nrf2 and its repressor protein Keap1 act as major regulators of cellular redox levels, which is attributed in part to the activation of Notch signaling pathway [16, 18]. Therefore, we investigated the effect of MCUR1 expression on Nrf2/Notch1 pathway in HCC cells. As shown in Fig. 4a, MCUR1 knockdown significantly decreased the nuclear translocation of Nrf2, which can be reversed by H_2_O_2_. In contrast, MCUR1 overexpression induced Nrf2 nuclear translocation, which can be abolished by ROS scavenger MitoTEMPO. To address downstream mediators of Nrf2, we observed the Notch pathway to be significantly changed. Our data showed that MCUR1 knockdown significantly decreased the expression of Notch1 in the cytoplasm and its active form NICD1 in nucleus of HCC cells, while MCUR1 overexpression had an opposite effect on Notch1 and NICD1 expression (Fig. 4c and d). Furthermore, H_2_O_2_ significantly reversed MCUR1 knockdown-mediated Notch1 down-regulation, whereas scavenging ROS by MitoTEMPO considerably inhibited the MCUR1 overexpression-mediated Notch1 activation (Fig. 4c and d). As expected, the MCUR1 knockdown-mediated Notch1 inhibition was significantly reversed by treatment with Oltipraz (OPZ), which is an activator of Nrf2. However, knockdown of Nrf2 by siRNA considerably inhibited the MCUR1 overexpression-mediated Notch1 activation (Fig. 4c and d and Additional file 4: Figure S3a).

### Activation of ROS/Nrf2/Notch pathways was essential for MCUR1-induced EMT and HCC metastasis

**Fig. 5:**
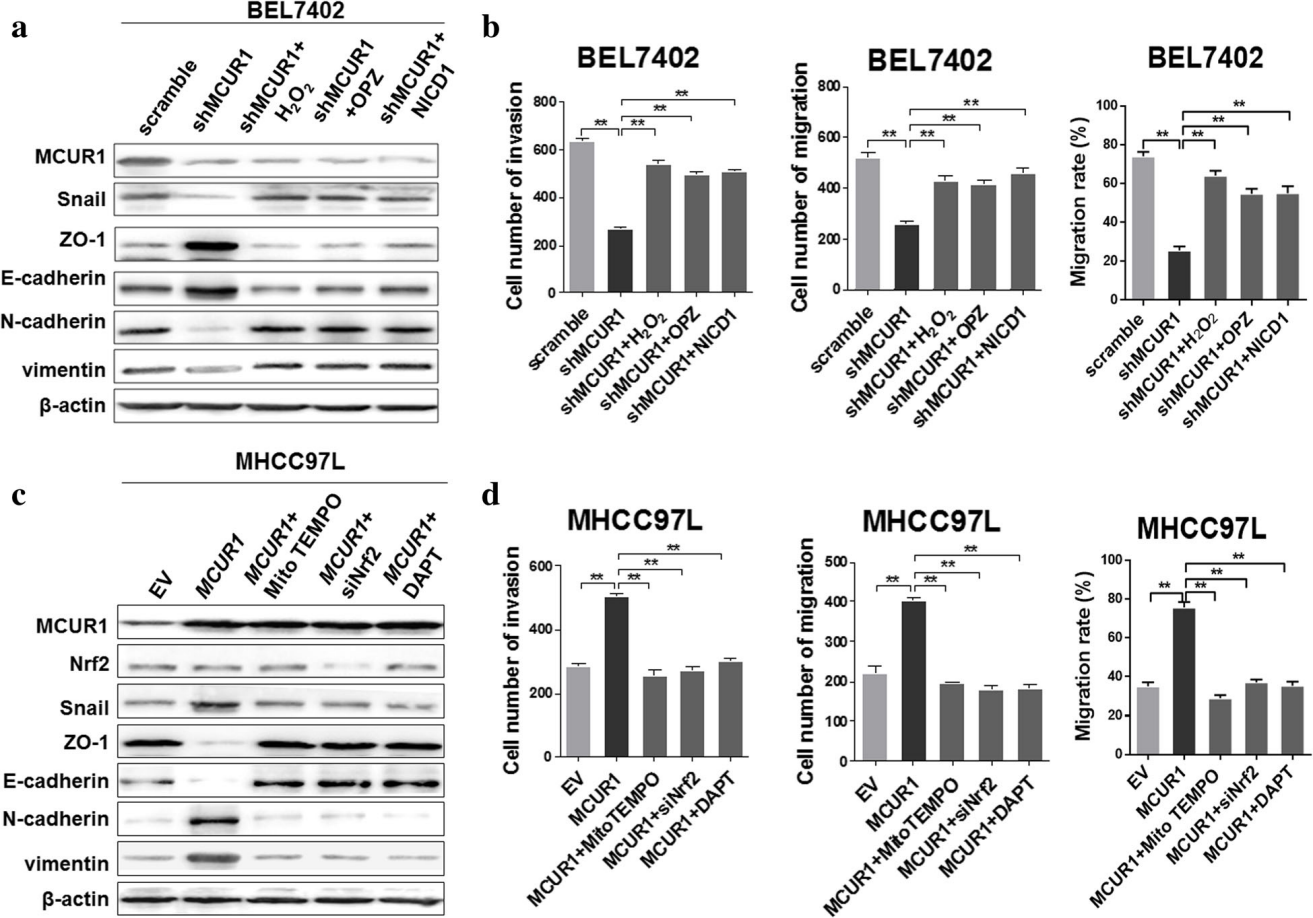
Activation of ROS/Nrf2/Notch pathways is essential for MCUR1-induced EMT and HCC metastasis. **a**, **c** Western blot analysis for expression levels of Snail, ZO-1, E-cadherin, N-cadherin and Vimentin in stably transfected HCC cells with treatments as indicated. **b**, **d** Transwell assay for migration and invasion ability and wound healing assays for migration rate in stably transfected HCC cells with treatment as indicated. H_2_O_2_ (25 μM, for 1 h), OPZ (20 μM, for 1 h), MitoTEMPO (5 μM for 1 h), DAPT (2 μM, for 6 h); cells were transfected with siRNA or expression vector for 48 h. NICD1, NICD1 expression vector; siNrf2, siRNA against Nrf2. Migration rate% = (Migrated distance at indicated time - initial distance) / (initial distance) × 100%. Data shown are the mean ± SD from three independent experiments. * *P* < 0.05; ** *P* < 0.01

We further investigated the functions of ROS/Nrf2/Notch pathway in MCUR1-induced EMT and invasion of HCC cells. Western blot analysis demonstrated that treatment with H_2_O_2,_ OPZ or NICD1 significantly reversed the MCUR1 knockdown-mediated epithelial transition, as indicated by increased expression of ZO-1, E-cadherin and decreased expression of N-cadherin, Vimentin and Snail (Fig. 5a). Moreover, transwell and scratch assays demonstrated that treatment with H_2_O_2_, OPZ and NICD1 significantly reversed the MCUR1 knockdown-mediated inhibition of HCC cell migration and invasion (Fig. 5b and Additional file 5: Figure S4a, b and c). In contrast, when treated with MitoTEMPO, siNrf2 or Notch1 inhibitor DAPT, the MCUR1 overexpression-mediated mesenchymal transition was significantly reversed, as indicated by decreased expression of ZO-1 and E-cadherin and increased expression of N-cadherin, Vimentin and Snail (Fig. 5c). Simultaneously, MCUR1 overexpression-mediated enhancement of HCC cell migration and invasion was significantly reversed by MitoTEMPO, siNrf2 or DAPT (Fig. 5d and Additional file 5: Figure S4d, e and f).

### Mitochondrial calcium buffering inhibited ROS/Nrf2/Notch pathway and MCUR1-induced EMT and HCC metastasis

**Fig. 6:**
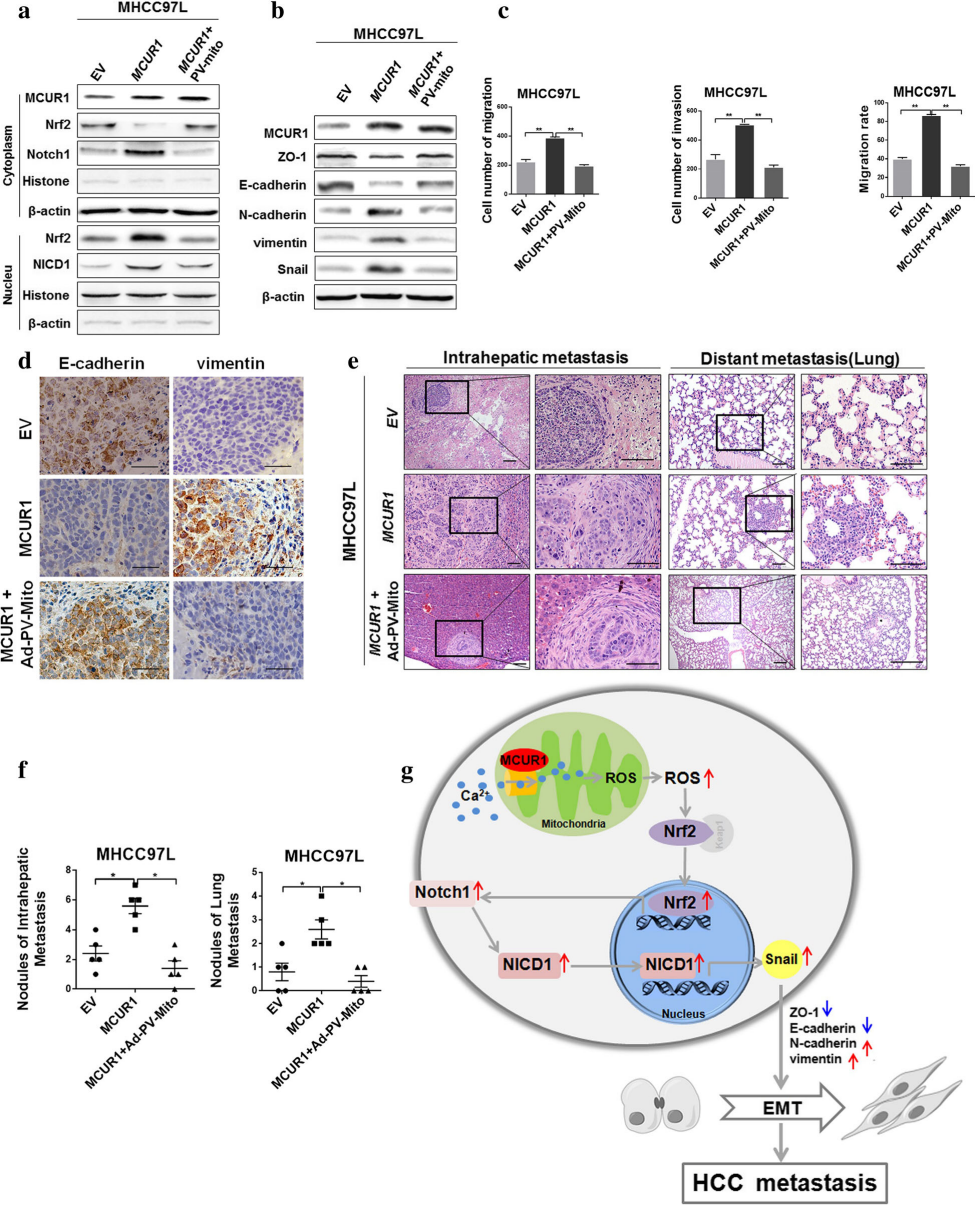
Mitochondrial calcium buffering inhibits ROS/Nrf2/Notch pathway and MCUR1-induced EMT and HCC metastasis. **a** Western blot analysis for expression levels of Nrf2, Notch1and NICD1 in stably transfected HCC cells with treatments as indicated. Mitochondrial Ca^2+^ was buffered by transient transfection of expression vector encoding parvalbumin with mitochondria target sequence (PV-Mito) for 48 h, where appropriate. **b** Western blot analysis for expression levels of ZO-1, E-cadherin, N-cadherin, Vimentin and Snail in stably transfected HCC cells with treatments as indicated. **c** Transwell assays for migration and invasion ability and wound healing assays for migration rate in stably transfected HCC cells with treatment as indicated. **d** Representative immunohistochemical staining images of E-cadherin and Vimentin in EV, MCUR1 and the Ad-PV-Mito groups (injection of PV-Mito adenovirus into tail vein). **e** Histological analyses of intrahepatic and lung metastatic nodules in EV, MCUR1 and the Ad-PV-Mito group (injection of PV-Mito adenovirus into tail vein). Images showing representative H&E staining of liver and lung tissue samples from the different experimental groups (*n* = 5 /group). **f** The number of intrahepatic and lung metastasis nodules was quantified in H.E. sections. **g** The schematic for mechanism underlying epithelial-mesenchymal transition and metastasis facilitated by MCUR1. Data shown are the mean ± SD from three independent experiments. * *P* < 0.05; ** *P* < 0.01

Our results had shown that the scavenging of [Ca^2+^] _m_ by PV-Mito considerably decreased the mitochondrial Ca^2+^, total ROS and mROS in HCC cells (Additional file 6: Figure S5a, b, c and d). We also shown that PV-Mito considerably inhibited MCUR1-mediated activation of Nrf2/Notch pathway (Fig. 6a). Moreover, we found that PV-mito significantly reversed the MCUR1-mediated decreased expression of ZO-1 and E-cadherin and increased expression of N-cadherin, Vimentin and Snail (Fig. 6b). MCUR1-mediated increase of migration and invasion was also reversed significantly by PV-Mito (Fig. 6c and Additional file 6 Figure S5e, f and g).

We further tested the effect of calcium buffering on HCC metastasis in xenograft models. IHC assays demonstrated that PV-Mito-recombined adenovirus (Ad-PV-Mito) treatment significantly inhibited the mesenchymal transition of MCUR1 overexpressed HCC cells in nude mice (Fig. 6d). Simultaneously, intrahepatic metastasis and lung metastasis were also remarkably repressed in Ad-PV-Mito group when compared with the control group (Fig. 6e and f).

## Discussion

Previous studies have demonstrated the important role of MCUR1 in HCC cell survival. Similar with previous reports, our study further confirmed the clinical significance of MCUR1 in EMT and HCC metastasis. More importantly, we provided the first evidence indicating high MCUR1 expression in HCC tissues with metastasis and significant association between MCUR1 expression and tumor progression in HCC patients. Moreover, the in vitro and in vivo experiments also first demonstrated that MCUR1-induced mitochondrial Ca^2+^ uptake activated the ROS/Nrf2/Notch signaling and thus facilitated the epithelial-mesenchymal transition and metastasis in hepatocellular carcinoma (Fig. 6g). Thus, MCUR1 has a great potential to be used as a prognosis marker or therapy target in clinical management of HCC patients.

EMT is closely involved in a series of pathological processes of HCC progression, including invasion, metastasis and chemoresistance. High expression of mesenchymal markers often indicates worse prognosis in HCC patients. EMT is regulated by several key transcriptional factors including Snail (zinc finger proteins Snail and Slug), Zeb (zinc finger and homeodomain proteins Zeb1 and Zeb2) and Twist (Twist1, Twist2) [19]. In the present study, we for the first time demonstrated that MCUR1 expression was significantly associated with the EMT of HCC cells both in vitro and in vivo. Moreover, MCUR1 knockdown and overexpression caused the expression change of Snail instead of Slug, suggesting that MCUR1 may promote the EMT of HCC cells through regulating Snail. Simultaneously, we found that MCUR1 expression was significantly associated with the metastasis of HCC, and MCUR1 expression level significantly affected the invasion in vitro and metastasis in vivo of HCC cells. These data suggest that MCUR1 expression may contribute to the metastasis of HCC by promoting EMT.

Ca^2+^ is the most abundant second messenger in human cells and has a substantial diversity of roles in fundamental cellular physiology, including gene expression, cell cycle control, cell motility, autophagy and apoptosis. Disruption of normal Ca^2+^ signaling contributes to the development of malignant phenotypes. There has been an increasing awareness that tumorigenic pathways are associated with altered expression level or abnormal activation of Ca^2+^ channels or transporters. Previous studies have demonstrated that abnormal Ca^2+^ signaling is involved in the proliferation, adhesion, migration, invasion and EMT of cancer cells [20]. Intracellular Ca^2+^ homeostasis is largely regulated by mitochondria mainly through MCU complex and its regulators, such as MCUR1. Previous studies have demonstrated that the dysregulation of mitochondrial Ca^2+^ uptake-associated proteins contributes to the survival, proliferation, metastasis and chemoresistance of cancer cells. MCUR1 is a critical component of MCU complex which is required for mitochondrial Ca^2+^ uptake and maintenance of normal cellular bioenergetics. MCUR1 silencing resulted in a dramatic reduction in the [Ca^2+^]mito (− ~ 85% in Rhod-2 fluorescence compared to the control) without modifying the cytosolic Ca^2+^ content [21]. However, its biological roles in cancer development remain largely unclear. Our previous studies have demonstrated that MCUR1 expression is frequently upregulated in HCC and MCUR1-mediated Ca^2+^ signaling promotes HCC cell survival. In this study, our findings that chelating mitochondrial Ca^2+^ by PV-Mito significantly suppressed MCUR1 overexpression-induced EMT and invasion of HCC cells further indicate that mitochondrial Ca^2+^ signaling mediates MCUR1-induced HCC metastasis.

Mitochondria are an essential source of reactive oxygen species in most mammalian cells. [Ca^2+^]_m_ has been reported to play an important role in the generation of ROS. Our previous studies have demonstrated that the overexpression of either MCU or MCRU1 promotes the production of ROS and oxidative status in HCC cells. ROS play a key role in many intracellular signaling pathways which contributes to the tumorigenesis. Nrf2 as the major effector of ROS in human cells regulates the expression of more than 100 genes, including Notch1 [18]. Upon oxidative stress, Nrf2 is detached from its inhibitor Keap-1 and translocated to the nucleus to promote the transcription of target genes by binding to promoter regions [22, 23]. Currently, growing evidences suggest that constitutive upregulation of Nrf2 is linked to cancer development, progression and resistance to radiotherapy. Moreover, Nrf2 promotes the invasion of cancer cells and contributes to poor prognosis of patients. In this study, we found that similar like H_2_O_2_, overexpression of MCUR1 had a remarkable effect to induce the Nrf2 nuclear translocation and Notch1 activation, which can be abolished by ROS scavenger. Moreover, Notch1 activation can be suppressed by either Nrf2 silencing or H_2_O_2_ scavenging, suggesting that ROS/Nrf2/Notch1 axis may act as the downstream signaling of MCUR1-induced Ca^2+^ homeostasis remodeling.

Notch signaling is a critical regulator in embryo development through the modulation of cell–cell communication [24]. Increasing evidences indicate that Notch signaling plays important roles in cancer development and progression [25, 26]. Elevated Notch1 expression has been observed in a series of malignancies including HCC and is commonly associated with aggressive cancer phenotypes. Notch signaling has been shown to promote EMT of cancer cells [27, 28]. Previous studies have demonstrated that the inhibition of Notch signaling by DAPT leads to the decrease of EMT in HCC cell lines, whereas activation of Notch signaling by Jagged1 promotes the increase of EMT [29–31]. In this study, we found that MCUR1 overexpression promoted the activation of Notch1 signaling, which can be inhibited by Ca^2+^ chelating, ROS scavenging, or Nrf2 silencing. Moreover, MCUR1-induced EMT as well as Snail transcription can be suppressed by Nrf2 silencing or Notch1 inhibitor DAPT. These findings suggest that MCUR1 facilitated HCC EMT, invasion and migration mainly through Nrf2/Notch1 signaling activation.

## Conclusions

In summary, our study first confirmed the clinical significance of MCUR1 in HCC metastasis. In addition, we demonstrated that the essential role of MCUR1 in promoting EMT, invasion and migration through the activation of ROS/Nrf2/Notch signaling by inducing mitochondrial Ca^2+^ uptake. Therefore, our study suggests that MCUR1 may be a potential target in HCC treatment.

## Additional files

Additional file 1: **Table S1.** Primary antibodies used for immunohistochemistry and western blot. **Table S2.** Sequence of primers. (DOCX 20 kb)

Additional file 2: **Figure S1 related to Figure 1. a** Phase-contrast photographs showing the morphology of HCC cells before and after stable transfection with MCUR1 expression vector. **b** Representative confocal microscope images of mitochondrial Ca^2+^ levels ([Ca^2+^]_m_) detected using mitopericam in HCC cells. Scale bar: 20 μm. **c** Representative time-course recording of mitochondrial Ca^2+^ fluorescence detected using mitopericam. After 30-s baseline recording, [Ca^2+^]_m_ responses to 10 μM histamine in HCC cells was investigated. Ca^2+^ response signals were presented as maximal amplitude fluorescence intensity, which was defined as the maximal change of [Ca^2+^]_m_ relative to the basal [Ca^2+^]_m_. **d** mROS levels were analyzed by confocal microscope after staining with MitoSOX (4 μM) for 10 min in HCC cells. Representative confocal microscope images were presented. Scale bar: 20 μm. **e** Intracellular ROS levels were stained with fluorescence dye DCFH-DA then analyzed by flow cytometry in HCC cells. **f** Western blot analysis of Snail level in MHCC97L cells transiently transfected with siRNA, and in BEL7402 cells transiently transfected with expression vector. Scramble: vector encoding control shRNA; shMCUR1 vectors encoding short hairpin RNA (shRNA) against MCUR1. EV: Empty Vector; MCUR1: expression vectors encoding MCUR1; Snail, Snail expression vector; siSnail, siRNA against Snail. Data shown are the mean ± SD from three independent experiments. *P < 0.05; **P < 0.01. (JPG 5515 kb)

Additional file 3: **Figure S2 related to Figure 2. a** Histological analyses of intrahepatic and lung metastatic nodules from HCC metastasis nude mice model by hematoxylin and eosin (H&E) staining. Images showing representative H&E staining of liver and lung tissue samples from the different experimental groups (*n* = 5 /group). Snail adenovirus and shSnail adenovirus was injected by tail vein. **b** The number of intrahepatic and lung metastasis nodules was quantified in H.E. sections. **c** Western blot analysis for indicated markers was performed with liver tissue lysates from three representative mice per group. Data shown are the mean ± SD from three independent experiments. * *P* < 0.05; ** *P* < 0.01. (JPG 3671 kb)

Additional file 4: **Figure S3 related to Figure 4. a** Western blot analysis of Nrf2 level in MHCC97L cells transiently transfected with siRNA. (JPG 52 kb)

Additional file 5: **Figure S4 related to Figure 5. a**, **d** Transwell assay for invasion and **b**, **e** migration ability of HCC cells with treatment as indicated. **c**, **f** Wound healing assays for migration rate in HCC cells with treatment as indicated. (JPG 9852 kb)

Additional file 6: **Figure S5 related to Figure 6. a** Co-localization of the mitochondria labeled with Mito-tracker (Red) and mitochondrial Ca^2+^ detected by mitopericam (Green) in MHCC97L cells. Scale bar: 20 μm. **b** Representative traces and quantification of [Ca^2+^]_m_ in HCC cells PV protein with mitochondrial translocation signal was used to buffer mitochondrial Ca^2+^. **c** Mitochondrial ROS levels were analyzed by confocal microscope after staining with MitoSOX (4 μM) for 10 min in HCC cells with treatment as indicated. Representative confocal microscope images were presented. Scale bar: 20 μm. Mitochondrial Ca^2+^ was buffered by transient transfection of expression vector encoding parvalbumin with mitochondria target sequence (PV-Mito) for 48 h, where appropriate. **d** Intracellular ROS levels were analyzed by flow cytometry after staining with fluorescence dye DCFH-DA in HCC cells with treatment as indicated. **e** Transwell assay for invasion and **f** migration ability of HCC cells with treatment as indicated. **g** Wound healing assays for migration rate in HCC cells with treatment as indicated. Data shown are the mean ± SD from three independent experiments. ** *P* < 0.01. (JPG 7415 kb)

## Abbreviations

[Ca^2+^]m: Mitochondrial Ca^2+^
EMT: Epithelial-mesenchymal transition
HCC: Hepatocellular carcinoma
IF: Immunofluorescence
IHC: Immunohistochemical
KEAP1: Kelch-like ECH-associated protein 1
MCU: Mitochondrial calcium uniporter
MCUR1: Mitochondrial calcium uniporter regulator 1
MICU1: Mitochondrial calcium uptake 1
mitoROS: mitochondrial reactive oxygen species
Nrf2: Nuclear factor erythroid-2 related factor 2
OPZ: Oltipraz
ROS: Reactive oxygen species
TNM: Tumor Node Metastasis
ZO-1: Zonula occludens-1
