# Supplementary material for: MCUR1 facilitates epithelial-mesenchymal transition and metastasis via the mitochondrial calcium dependent ROS/Nrf2/Notch pathway in hepatocellular carcinoma
Source: J Exp Clin Cancer Res. 2019 Mar 25;38:136. doi: 10.1186/s13046-019-1135-x (PMC6434841; doi:10.1186/s13046-019-1135-x)
Supplement: Supplementary file 1 — Table S1. Primary antibodies used for immunohistochemistry and western blot. Table S2. Sequence of primers. (DOCX 20 kb) [file 13046_2019_1135_MOESM1_ESM.docx]

**Table S1. Primary antibodies used for immunohistochemistry and western blot.**

| **Antibody** | **Company (Cat. No.)** | **Working dilutions** |
| --- | --- | --- |
| MCUR1 | SIGMA (HPA055189) | WB: 1/250 IHC: 1/250 |
| ZO-1 | Proteintech (66452-1-AP) | WB: 1/500 IF:1/50 |
| E-cadherin | Cell Signaling (#3195) | WB: 1/500 IF:1/50 IHC: 1/50 |
| N-cadherin | Proteintech (22018-1-AP) | WB: 1/500 IF:1/50 |
| Vimentin | Cell Signaling (#5741) | WB: 1/1000IF:1/100 IHC: 1/50 |
| Snail | Proteintech (13099-1-AP) | WB: 1/500 |
| Slug | Proteintech (12129-1-AP) | WB: 1/200 |
| Notch1 | Abcam (ab52627) | WB: 1/1000 |
| NICD1 | Abcam (ab8925) | WB: 1/1000 |
| Nrf2 | Proteintech (16396-1-AP) | WB: 1/500 |

**Table S2. Sequence of primers**

1. **Primers used in gene cloning**

| MCUR1 | Forward primer | GGGAATTCTATGGACTGCGGCTCGGT |
| --- | --- | --- |
|  | reverse primer | GGGGTACCTTAGATCCACAGGCGATA |
| Parvalbumin (PV) | forward primer | CGCGGATCCATGTCGATGACAGACTTGCTGAACG |
|  | reverse primer | CGGACCGGTGCTTTCAGCCACCAGAGTGGAGAATTC |

1. **siRNA**

| NRF2 siRNA | sense | GCUUUUGGCGCAGACAUUC |
| --- | --- | --- |
|  | antisense | GAAUGUCUGCGCCAAAAGC |
| Snail siRNA | sense | AGCGAGCUGCAGGACUCTA |
|  | antisense | UAGAGUCCUGCAGCUCGCU |
