# Supplementary figures and images for: MCUR1 facilitates epithelial-mesenchymal transition and metastasis via the mitochondrial calcium dependent ROS/Nrf2/Notch pathway in hepatocellular carcinoma
Source: J Exp Clin Cancer Res. 2019 Mar 25;38:136. doi: 10.1186/s13046-019-1135-x (PMC6434841; doi:10.1186/s13046-019-1135-x)

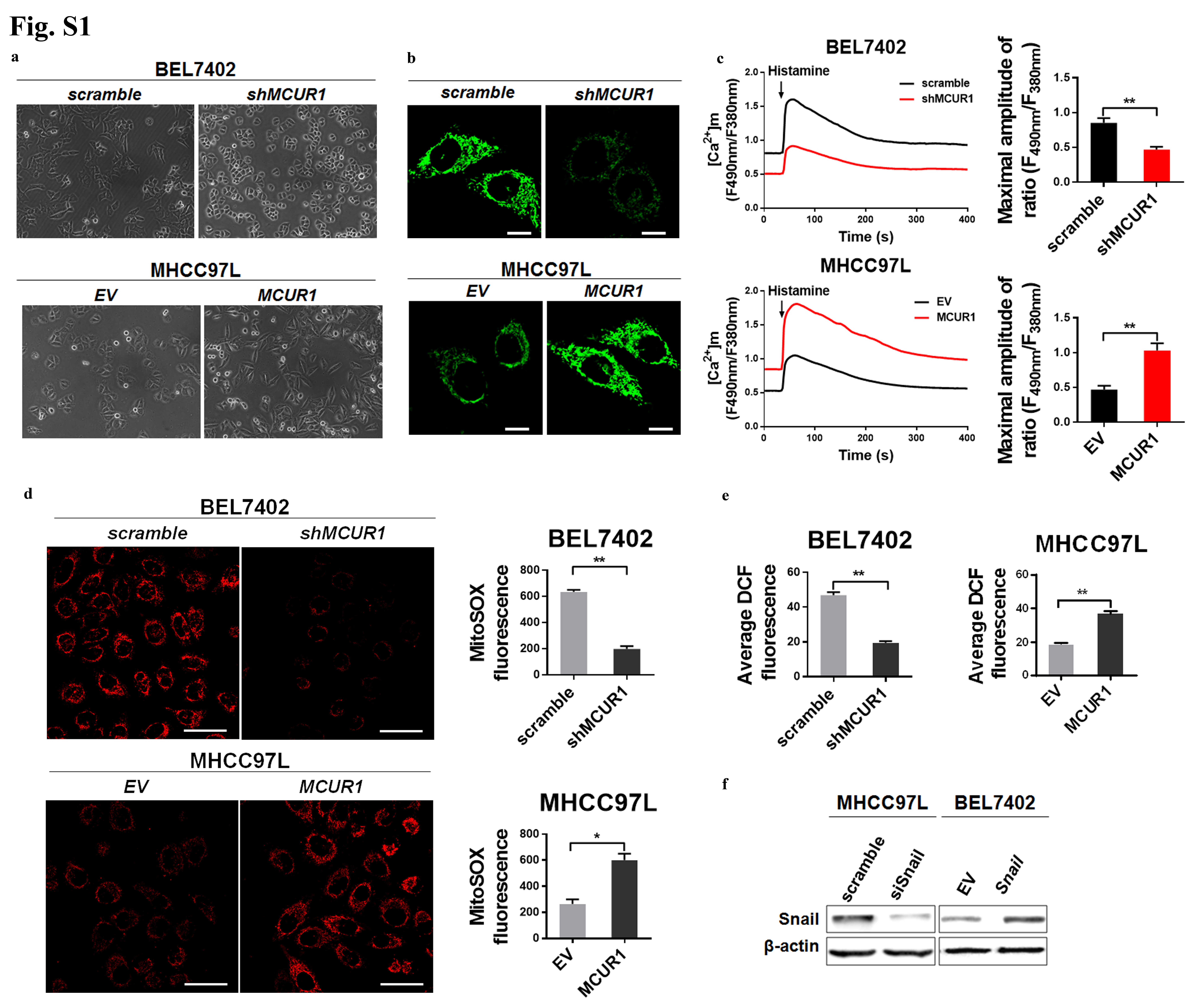

Supplement: Supplementary file 2 — Figure S1 related to Figure 1. a Phase-contrast photographs showing the morphology of HCC cells before and after stable transfection with MCUR1 expression vector. b Representative confocal microscope images of mitochondrial Ca2+ levels ([Ca2+]m) detected using mitopericam in HCC cells. Scale bar: 20 μm. c Representative time-course recording of mitochondrial Ca2+ fluorescence detected using mitopericam. After 30-s baseline recording, [Ca2+]m responses to 10 μM histamine in HCC cells was investigated. Ca2+ response signals were presented as maximal amplitude fluorescence intensity, which was defined as the maximal change of [Ca2+]m relative to the basal [Ca2+]m. d mROS levels were analyzed by confocal microscope after staining with MitoSOX (4 μM) for 10 min in HCC cells. Representative confocal microscope images were presented. Scale bar: 20 μm. e Intracellular ROS levels were stained with fluorescence dye DCFH-DA then analyzed by flow cytometry in HCC cells. f Western blot analysis of Snail level in MHCC97L cells transiently transfected with siRNA, and in BEL7402 cells transiently transfected with expression vector. Scramble: vector encoding control shRNA; shMCUR1 vectors encoding short hairpin RNA (shRNA) against MCUR1. EV: Empty Vector; MCUR1: expression vectors encoding MCUR1; Snail, Snail expression vector; siSnail, siRNA against Snail. Data shown are the mean ± SD from three independent experiments. *P < 0.05; **P < 0.01. (JPG 5515 kb) [file 13046_2019_1135_MOESM2_ESM.jpg]

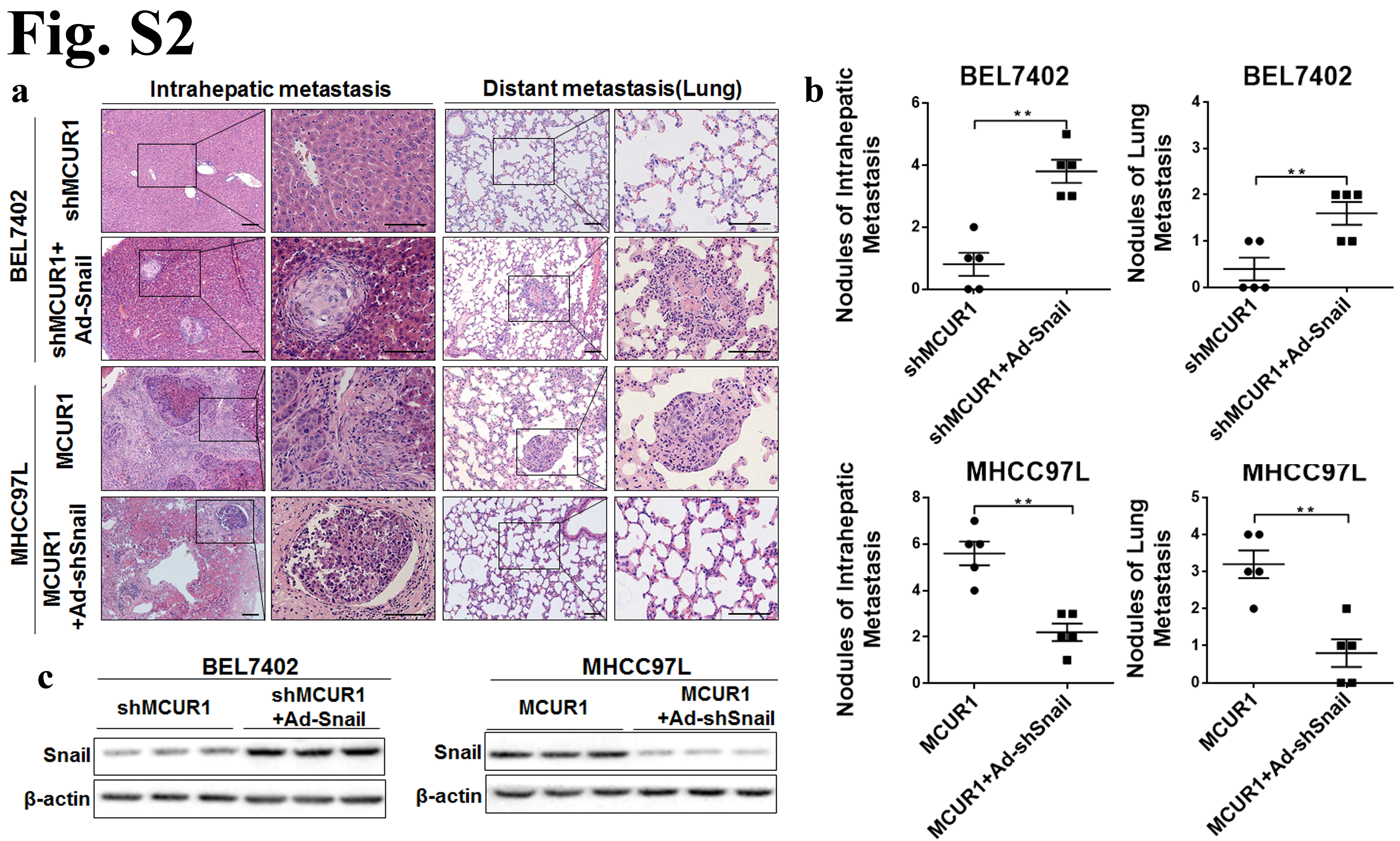

Supplement: Supplementary file 3 — Figure S2 related to Figure 2. a Histological analyses of intrahepatic and lung metastatic nodules from HCC metastasis nude mice model by hematoxylin and eosin (H&E) staining. Images showing representative H&E staining of liver and lung tissue samples from the different experimental groups (n = 5 /group). Snail adenovirus and shSnail adenovirus was injected by tail vein. b The number of intrahepatic and lung metastasis nodules was quantified in H.E. sections. c Western blot analysis for indicated markers was performed with liver tissue lysates from three representative mice per group. Data shown are the mean ± SD from three independent experiments. * P < 0.05; ** P < 0.01. (JPG 3671 kb) [file 13046_2019_1135_MOESM3_ESM.jpg]

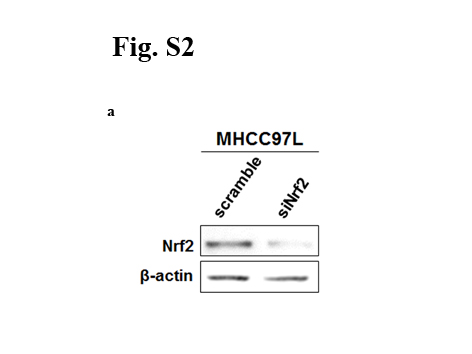

Supplement: Supplementary file 4 — Figure S3 related to Figure 4. a Western blot analysis of Nrf2 level in MHCC97L cells transiently transfected with siRNA. (JPG 52 kb) [file 13046_2019_1135_MOESM4_ESM.jpg]

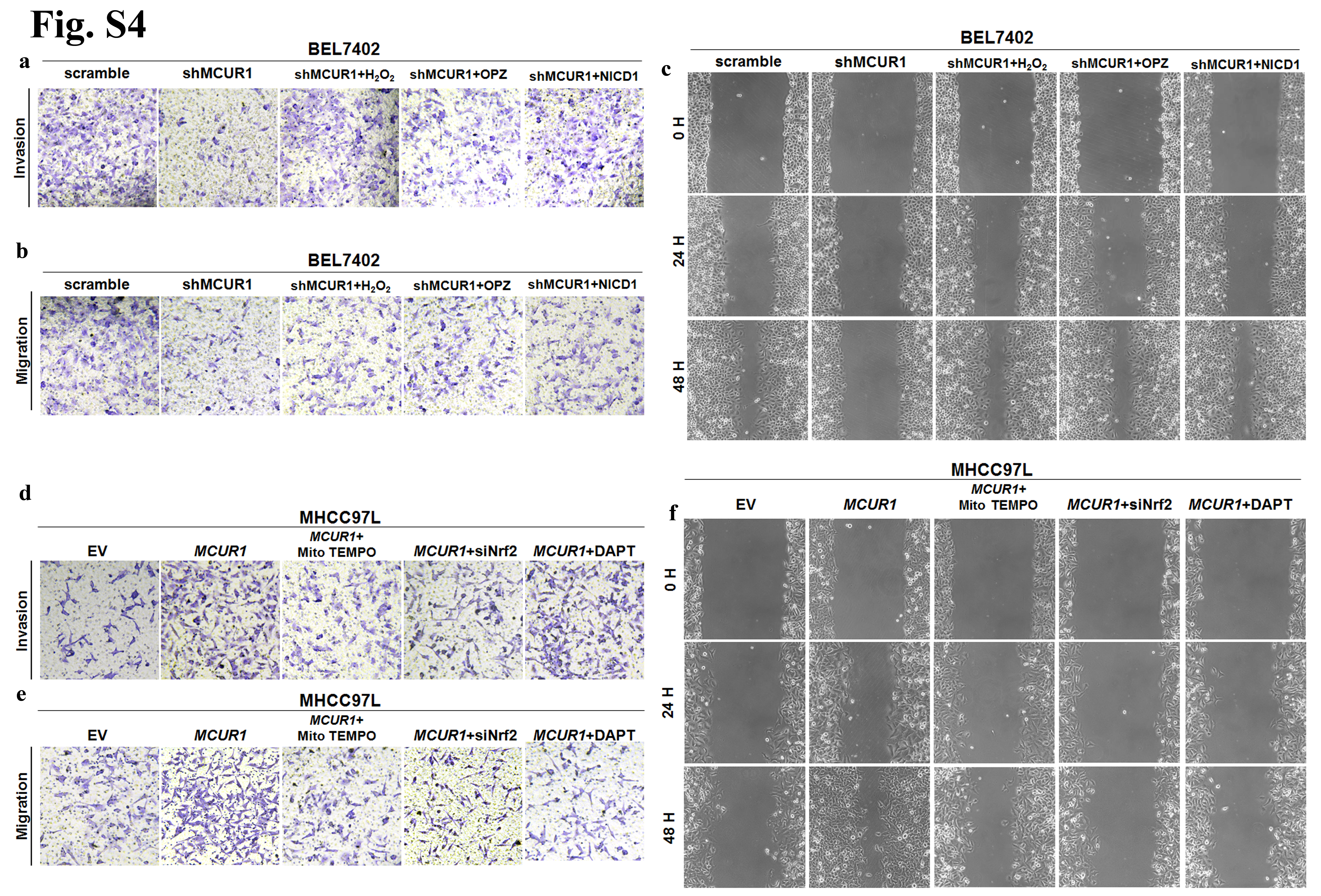

Supplement: Supplementary file 5 — Figure S4 related to Figure 5. a, d Transwell assay for invasion and b, e migration ability of HCC cells with treatment as indicated. c, f Wound healing assays for migration rate in HCC cells with treatment as indicated. (JPG 9852 kb) [file 13046_2019_1135_MOESM5_ESM.jpg]

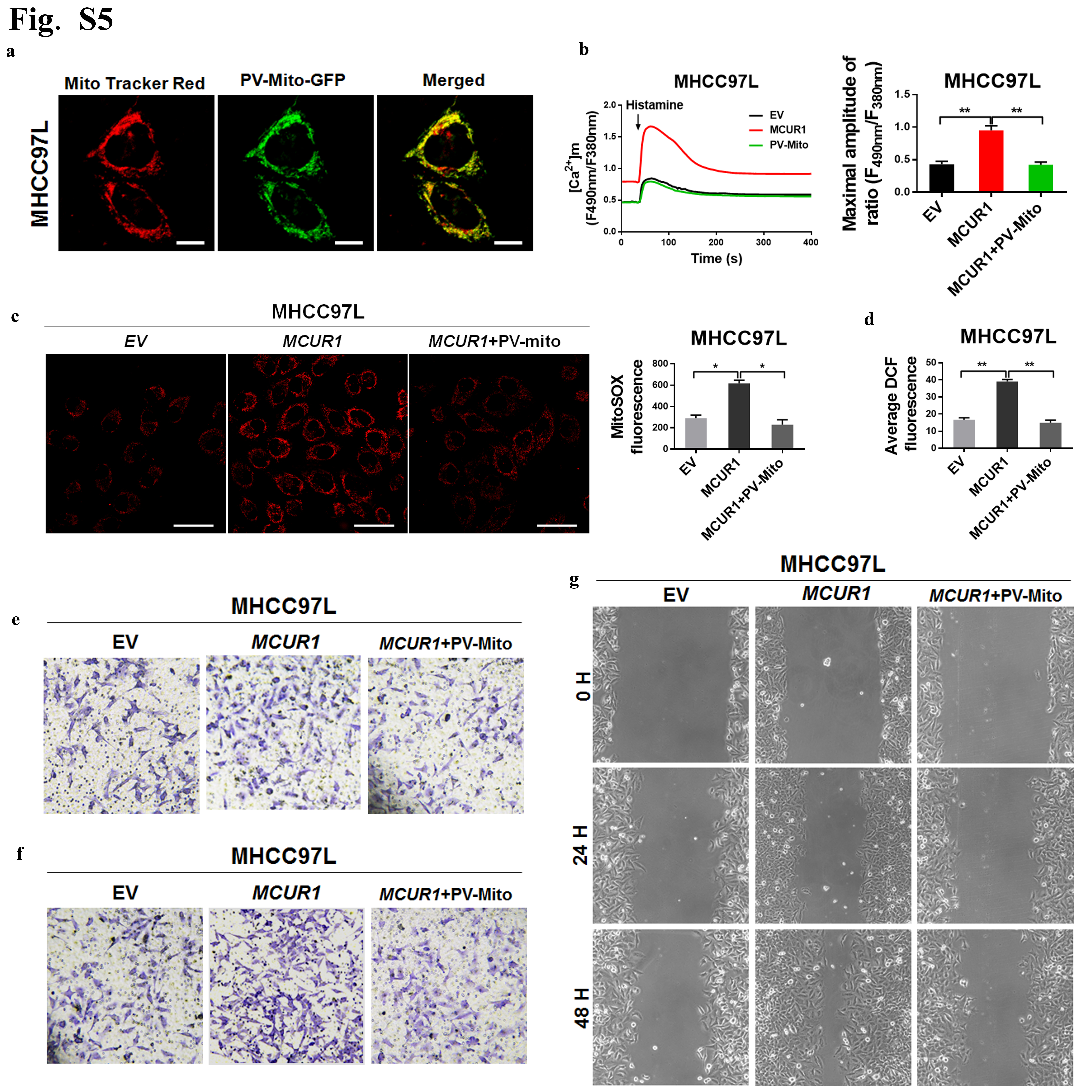

Supplement: Supplementary file 6 — Figure S5 related to Figure 6. a Co-localization of the mitochondria labeled with Mito-tracker (Red) and mitochondrial Ca2+ detected by mitopericam (Green) in MHCC97L cells. Scale bar: 20 μm. b Representative traces and quantification of [Ca2+]m in HCC cells PV protein with mitochondrial translocation signal was used to buffer mitochondrial Ca2+. c Mitochondrial ROS levels were analyzed by confocal microscope after staining with MitoSOX (4 μM) for 10 min in HCC cells with treatment as indicated. Representative confocal microscope images were presented. Scale bar: 20 μm. Mitochondrial Ca2+ was buffered by transient transfection of expression vector encoding parvalbumin with mitochondria target sequence (PV-Mito) for 48 h, where appropriate. d Intracellular ROS levels were analyzed by flow cytometry after staining with fluorescence dye DCFH-DA in HCC cells with treatment as indicated. e Transwell assay for invasion and f migration ability of HCC cells with treatment as indicated. g Wound healing assays for migration rate in HCC cells with treatment as indicated. Data shown are the mean ± SD from three independent experiments. ** P < 0.01. (JPG 7415 kb) [file 13046_2019_1135_MOESM6_ESM.jpg]
